# BDK Deficiency in Cerebral Cortex Neurons Causes Neurological Abnormalities and Affects Endurance Capacity

**DOI:** 10.3390/nu12082267

**Published:** 2020-07-29

**Authors:** Anna Mizusawa, Ayako Watanabe, Minori Yamada, Rina Kamei, Yoshiharu Shimomura, Yasuyuki Kitaura

**Affiliations:** 1 Laboratory of Nutritional Biochemistry, Department of Applied Biosciences, Graduate School of Bioagricultural Sciences, Nagoya University, Nagoya, Aichi 464-8601, Japan; mizusawa.anchan.81@outlook.jp (A.M.); watanabe.ayako@i.mbox.nagoya-u.ac.jp (A.W.); mynority758@gmail.com (M.Y.); rina.kamei.9712@gmail.com (R.K.); 2 Department of Food and Nutritional Sciences, College of Bioscience and Biotechnology, Chubu University, Kasugai, Aichi 487-8501, Japan; shimomura@isc.chubu.ac.jp

**Keywords:** BCAA, BDK, neuron, endurance exercise

## Abstract

Branched-chain amino acid (BCAA) catabolism is regulated by its rate-limiting enzyme, branched-chain α-keto acid dehydrogenase (BCKDH), which is negatively regulated by BCKDH kinase (BDK). Loss of BDK function in mice and humans leads to dysregulated BCAA catabolism accompanied by neurological symptoms such as autism; however, which tissues or cell types are responsible for the phenotype has not been determined. Since BDK is highly expressed in neurons compared to astrocytes, we hypothesized that neurons are the cell type responsible for determining the neurological features of BDK deficiency. To test this hypothesis, we generated mice in which BDK deletion is restricted to neurons of the cerebral cortex (BDK^Emx1-KO^ mice). Although BDK^Emx1-KO^ mice were born and grew up normally, they showed clasped hind limbs when held by the tail and lower brain BCAA concentrations compared to control mice. Furthermore, these mice showed a marked increase in endurance capacity after training compared to control mice. We conclude that BDK in neurons of the cerebral cortex is essential for maintaining normal neurological functions in mice, and that accelerated BCAA catabolism in that region may enhance performance in running endurance following training.

## 1. Introduction

Leucine, valine, and isoleucine are branched-chain amino acids (BCAAs) and are considered essential amino acids for mammals. BCAAs exist as free amino acids and are abundant in proteins, providing approximately 35% of essential amino acids in indispensable muscle proteins [1]. Free BCAAs are used as building blocks in protein synthesis as well as energy sources and nitrogen donors to BCAA catabolic enzymes. BCAAs, especially leucine, also have signaling effects [2]. One of the most well-known signaling functions is stimulation of protein synthesis via the mammalian target of rapamycin complex (mTORC1) with hormonal signals [3].

Metabolism of BCAAs plays notable roles in the brain by directly or indirectly providing essential nutrients as well as serving as components of neurotransmitters, for example, glutamate or amine neurotransmitters including 5-hydroxytryptamine also known as monoamine serotonin [4]. BCAAs are transported into the brain through the blood–brain barrier, mostly using system L1 and to a lesser extent, other systems [5]. The enzymes for BCAA catabolism mediate BCAA homeostasis in the brain [6]. In the first step of the BCAA catabolic pathway, branched-chain aminotransferase (BCAT) reversibly transaminates BCAAs to α-ketoglutarate, generating branched-chain α-ketoacids (BCKAs) and glutamate, and then BCKAs are irreversibly decarboxylated by branched-chain α-ketoacid dehydrogenase (BCKDH) into branched-chain acyl-CoAs, which are eventually catabolized to acetyl-CoA or succinyl-CoA. BCKDH is the rate-limiting enzyme of the pathway and is activated by dephosphorylation via a mitochondrial localized 2C-type serine-threonine protein phosphatase and inactivated by BCKDH kinase (BDK) [2]. The transamination reaction of BCAAs in the brain contributes about one-third of the nitrogen in glutamate in vitro [7]. There are two BCAT isozymes by which the reversible transamination is cell-specific; the mitochondrial isozyme (BCATm) is predominant in astrocytes, while the cytosolic isozyme (BCATc) is localized in a neuron [8,9]. BCATm-deficient mice exhibit markedly altered metabolic parameters, including increased food intake, leanness, enhanced energy expenditure, and improved insulin sensitivity [10], but are exercise intolerant with markedly decreased endurance to exhaustion [11]. The mutations in BCATm have been reported in patients of hypervalinemia and hyperleucine-isoleucinemia with mild memory impairment [12]. On the other hand, BCATc-deficient mice show enhanced T cell activation [13], but their neurological abnormalities have not yet been reported.

Although BCATs are expressed in both neurons and astrocytes, BCAA oxidation after the second step occurs specifically in neuronal cells [8,9]. Inborn errors of the BCKDH complex subunits result in maple syrup urine disease [14], and recently, BDK was also identified as a gene associated with neurological symptoms such as autism, epilepsy, and intellectual disability in humans [15,16]. BDK null mice (BDK-KO mice) demonstrate neurological abnormalities and epileptic seizures [17], suggesting that regulation of BCAA oxidation via BCKDH activity is critical for normal neurological functions. However, little is known about the site in the complex brain system where specific regulation of BCAA oxidation accounts for neurological dysfunctions. Previously, we generated mice lacking BDK (unrestricted BCAA oxidation) specifically in skeletal muscle and heart (BDK^M-KO^ mice). These mice have a lower myofibrillar protein concentration in skeletal muscle and a lower level of mTORC1 activity when fed a low-protein diet [18]. They also have lower exercise endurance capacity after training [19], which can be restored and exceeded with BCAA supplementation [20]. BDK^M-KO^ mice do not show any obvious neurological dysfunction, and thus, tissues other than skeletal muscle and heart are thought to be responsible for the phenotype of BDK-KO mice. Most brain function including cognition, movement, and consciousness is due to activity of the cerebral cortex, where the expression of the BCKDH complex is high, especially in neurons [21,22]. Here, to elucidate which region in the brain is important for regulation of BCAA oxidation for maintaining neurological functions, we used mice expressing Cre under the control of the empty spiracles homolog 1 (Emx-1) locus [23] to generate BDK^Emx1-KO^ mice, which are deficient in BDK protein in neurons of the cerebral cortex.

## 2. Materials and Methods

### 2.1. Animal Experiments

All experimental animal procedures were approved by the Animal Care Committee of the Graduate School of Bioagricultural Sciences, Nagoya University (approved number: 2018031310) and carried out following the relevant guidelines. Generation of *Bckdk*-deficient (BDK-KO) and muscle-specific *Bckdk*-deficient (BDK^M-KO^) mice on the C57BL/6 background has been described previously [18]. Cerebral cortex neuron-specific *Bckdk*-deficient (BDK^Emx1-KO^) mice were generated by crossing BDK-floxed mice with mice expressing Cre recombinase under the control of the *Emx1* locus [23] which was provided by the RIKEN BioResource Research Center (BRC No. RBRC01345) through the National BioResource Project of the Ministry of Education, Culture, Sports, Science and Technology (MEXT)/ Japan Agency for Medical Research and Development (AMED). We used mice carrying homozygous BDK-floxed alleles as well as the heterozygous Emx1-Cre allele as BDK^Emx1-KO^ mice, and Emx-Cre-negative BDK-floxed homozygous mice as control mice.

All mice were given free access to water and standard chow diet (CE-2, CREA Japan, Tokyo, Japan) after weaning, and were then switched to an experimental diet (D12450J (20% protein), Research Diets, New Brunswick, NJ, USA) at 7 to 8 weeks of age. Mice were maintained at 23 ± 2 °C with a 12-h light/dark cycle. Body weight and food intake of mice were monitored weekly. At 20 weeks of age, all experimental mice (*n* = 6 per group) were killed under anesthesia with isoflurane (FUJIFILM Wako Pure Chemical, Osaka, Japan) after fasting for 8 h from 08:00 to 16:00. Blood samples were obtained from the posterior vena cava with a syringe to prepare post-heparin plasma. The brains were quickly removed, and the forebrain region was dissected. The gastrocnemius, plantaris, and soleus muscles, liver, heart, subcutaneous and visceral adipose tissues, and kidney were excised. The dissected tissues were rapidly frozen in liquid nitrogen and stored at −80 °C until analyses.

### 2.2. Western Blotting

Western blot analysis was conducted using antisera recognizing BCKDH (both the E1 and E2 subunits of the complex) or BDK as described previously [24,25]. In brief, frozen tissues were ground into powder in liquid nitrogen, homogenized in extraction buffer, and centrifuged at 15,000× *g* for 15 min at 4 °C. Protein concentrations were determined using the Bicinchoninic acid protein assay (Thermo Scientific, Waltham, MA, USA). Then, 40 μg proteins were resolved with sodium dodecyl sulfate-polyacrylamide gel electrophoresis, transferred onto a polyvinylidene fluoride membrane (Millipore, Billerica, MA, USA), and detected with Western blotting using a primary antibody. Blots were then incubated with horseradish peroxidase-conjugated secondary antibody (rabbit anti-mouse IgG for BDK, goat anti-rabbit IgG for the E1 and E2 subunits of BCKDH complex; Bio-Rad Laboratories, Hercules, CA, USA). Immunoblots were developed using enhanced chemiluminescence Western blotting detection reagents (GE Healthcare, Buckinghamshire, UK), and proteins were visualized and quantified with the AE6962 Light Capture system (ATTO, Tokyo, Japan).

### 2.3. Amino Acid Profiling

For amino acid profiling of the mouse forebrain, frozen tissues were ground into powder in liquid nitrogen, and 20–30 mg of the samples were used. For plasma BCAA profiling, 10 μL of plasma was used. Each sample was mixed with a solvent mixture (CH_3_OH:H_2_O:CHCl_3_, 2.5:1:1), followed by addition of 10 μL of 0.5 mg/mL 2-isopropylmalic acid (Sigma-Aldrich, St. Louis, MO, USA) and 10 μL of UL-^13^C,^15^N (Taiyo Nissan, Tokyo, Japan) as internal standards dissolved in distilled water. The mixture was incubated for 30 min at 37 °C in a shaking incubator and then centrifuged at 16,000× *g* for 3 min at 4 °C. The supernatant was dissolved in distilled water and centrifuged at 16,000× *g* for 3 min at 4 °C. The supernatant was evaporated to dryness.

As the first derivatizing agent, 40 μL of 20 mg/mL methoxyamine hydrochloride (Sigma-Aldrich) dissolved in pyridine was added and incubated for 90 min at 30 °C in a shaking incubator. The second derivatizing agent, 20 μL of N-methyl-N-trimethylsilyl-trifluoroacetamide (GL Science, Tokyo, Japan), was mixed and incubated for 30 min at 37 °C in a shaking incubator. The mixture was then centrifuged at 16,000× *g* for 3 min at 4 °C, and the resultant supernatant was transferred to a vial for gas chromatography/mass spectrometry (GC/MS) measurement. GC/MS was carried out using GCMS-TQ8040 (Shimadzu Co., Kyoto, Japan) with a DB-5 capillary column (30 m × 0.25 mm i.d.; 1 μm film thickness; Agilent J & W Scientific, Folsom, CA, USA) as described previously [26]. The inlet temperature was set at 250 °C, and the injection volume was 1 μL (splitless mode). The GC column temperature was programmed to remain at 100 °C for 4 min, followed by a 10 °C min^−1^ linear ramp to a final temperature of 320 °C, which was held for 11 min. Helium was used as a carrier gas at a flow rate of 1.1 mL min^−1^. The transfer line and ion source temperatures were maintained at 280 °C and 200 °C, respectively. For ionization, the electron impact mode at 70 eV was used. Argon gas was used as a collision-induced dissociation gas. Metabolites were detected using the Smart Metabolites Database (Shimadzu), which included the relevant multiple reaction monitoring (MRM) method file and data regarding the GC analytical conditions, MRM parameters, and retention index used for amino acid measurement following the report.

### 2.4. Assessment of Neurological Abnormalities

Neurological abnormalities were assessed by observing hind limb clasping as previously reported [17]. Mice (*n* = 5–8, males, 15 weeks of age per genotype) fed either 20% or 5% protein diet (CREA Japan Inc.) from 8 to 15 weeks old were suspended by the tail for 20 s to initiate clasping. The score was a simple grading scale from 0 to 3 (normal, 0; moderate hind limb clasping, 0.5; moderately severe hind limb clasping, 1; complete hind limb clasping, 2; complete hind limb clasping and trunk-curling posture, 3).

### 2.5. Endurance Exercise

Animals (*n* = 6–26, males, 8 to 15 weeks of age per genotype) were used to measure endurance exercise capacity. Before and after the 2-week training program described below, the mice were fasted for 12 h from 21:00 to 09:00 before the start of the endurance exercise session to obtain stable metabolic conditions. Mice were placed on an enclosed treadmill and then began running at 10 m min^−1^, and the speed was increased by 1 m min^−1^ every 4 min up to 20 m min^−1^ with a 10% incline. Mice were forced to run because an electric grid (voltage of 35 V) was placed at the end of the treadmill. Exhaustion was defined as mice remaining on the shock grid for 5 s five consecutive times. The 2-week training program was conducted using a treadmill with a 10% incline; mice ran for 1 h per day, 5 days per week. The running speed was 15 m min^−1^ during the first week and 18 m min^−1^ during the second week. An interval of 2 days was set between the weeks.

### 2.6. Statistical Analysis

Values represent the mean ± standard error of the mean (SEM) or percentage, as applicable. Statistical significance of differences between groups was measured using a two-tailed Student’s *t*-test or Mann–Whitney test (two groups) or Dunnett’s multiple comparisons test (four groups). The time for the endurance exercise capacity test was tabulated with the Kaplan–Meier method, and BDK-deficient groups were compared to control mice using the log-rank (Mantel–Cox) test. Differences were considered significant at *p* < 0.05. The analyses were conducted using StatView software version 5 (SAS Institute, Cary, NC, USA). 

## 3. Results

### 3.1. Characteristics of Mice with BDK Deficiency in Cerebral Cortex Neurons (BDK^Emx1-KO^ Mice)

**Figure 1:**
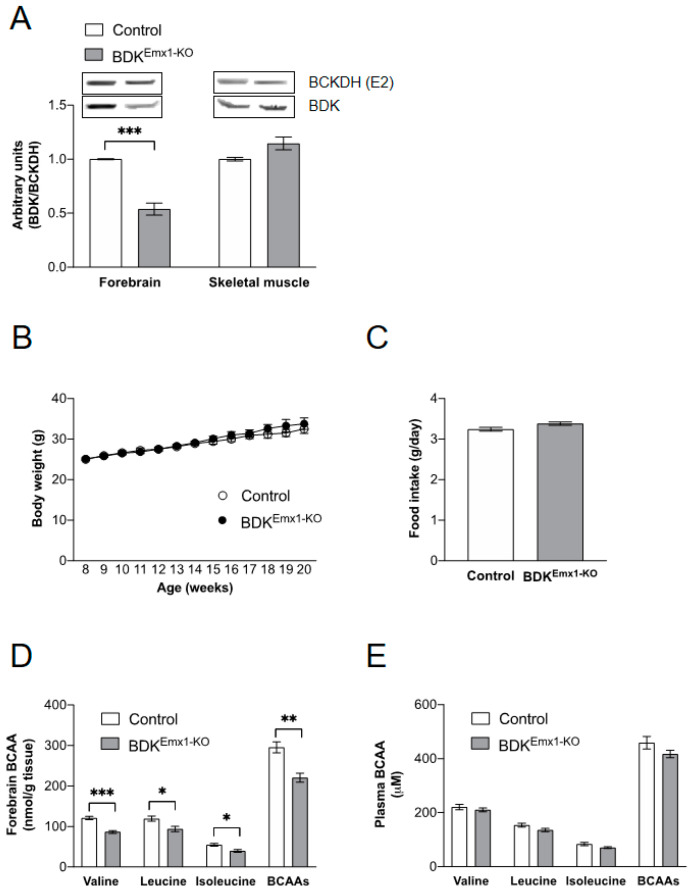
Characterization of BDK^Emx1-KO^ mice. BDK protein expression in the forebrain and skeletal muscle (**A**), growth curves (**B**), food intake (**C**), and BCAA concentrations in brain (**D**), and plasma (**E**) in control and BDK^Emx1-KO^ mice. Food intake is the average of weekly monitoring. Values represent the mean ± SEM (*n* = 3–6 per group). * *p* < 0.05, ** *p* < 0.01, and *** *p* < 0.001 compared with control values. Differences were determined using Student’s t-test. BCAA: branched-chain amino acid; BDK: BCKDH kinase; KO: knock-out.

We used mice that express Cre recombinase under control of the homeobox gene, Emx1, to delete BDK in cerebral cortex neurons [23]. As shown in Figure 1A, BDK protein expression was reduced in BDK^Emx1-KO^ mice by approximately 50% in the forebrain compared to that of the control. However, BDK expression in skeletal muscle was not changed (Figure 1A). In previous reports, neurological defects and growth retardation were found in global BDK-deficient (BDK-KO) mice, but not in muscle-specific BDK-deficient (BDK^M-KO^) mice [17,18]. Growth rate, food intake, and tissue weights were not significantly different between the control and BDK^Emx1-KO^ mice (Figure 1B,C; Table 1). Compared to control mice, concentrations of valine, leucine, and isoleucine were significantly reduced in the brain of BDK^Emx1-KO^ mice, and total BCAAs were approximately 25% lower (Figure 1D). Levels of Trp, Tyr, Phe, and Met, which share a transporter of system L1 for transport of BCAAs and other amino acids across the blood–brain barrier [4,5], did not differ between control and BDK^Emx1-KO^ mice in the brain (Table 2). Contrary to brain BCAAs, plasma BCAA concentrations were not altered in BDK^Emx1-KO^ compared to control mice (Figure 1E).

### 3.2. Neurological Abnormalities in BDK^Emx1-KO^ Mice

**Figure 2:**
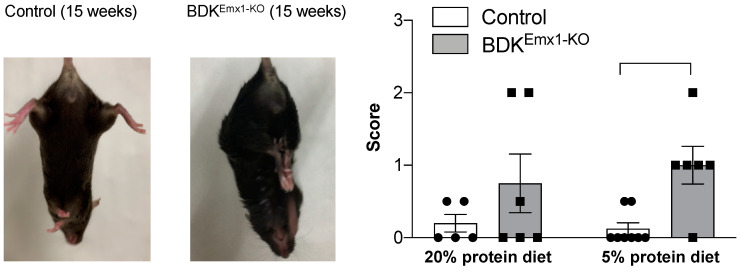
Neurological abnormalities in BDK^Emx1-KO^ mice. Representative hindlimb clasping in control and BDK^Emx1-KO^ mice, and the clasping scores of mice fed a 20% or 5% protein diet are shown. Values represent the mean ± SEM (*n* = 5–8 per group). ** *p* < 0.01. Individual results were indicated as a closed circle (Control mice) and a closed square (BDK^Emx1-KO^ mice). Differences were determined using the Mann–Whitney test. BCKDH: branched-chain α-keto acid dehydrogenase; BDK: BCKDH kinase; KO: knock-out.

BDK^Emx1-KO^ mice showed clinching of the hind limbs to the body when held by the tail at 15 weeks of age, whereas control mice did not show such clasping, indicating an abnormality in the central nervous system in BDK^Emx1-KO^ mice (Figure 2). The clasping score was not significantly different between groups fed a 20% protein diet. However, when mice were fed a low-protein diet (5%), BDK^Emx1-KO^ mice showed higher scores compared to control mice fed the same diet (Figure 2).

### 3.3. Endurance Exercise Capacity in BDK^Emx1-KO^ Mice

**Figure 3:**
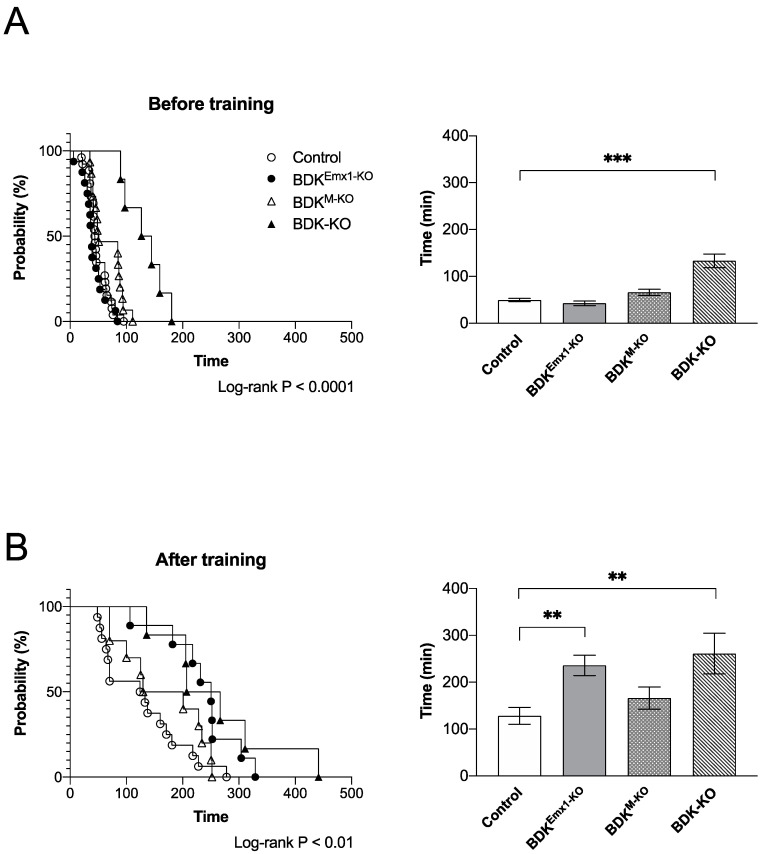
Endurance exercise performance in BDK^Emx1-KO^, BDK^M-KO^, and BDK-KO mice. Running time during endurance exercise of BDK^Emx1-KO^, BDK^M-KO^, and BDK-KO mice compared to control mice before (**A**) and after training (**B**). Values represent the mean ± SEM; before training, *n* = 26 for control, *n* = 16 for BDK^Emx1-KO^, *n* = 15 for BDK^M-KO^, and *n* = 6 for BDK-KO; after training, *n* = 16 for control, *n* = 9 for BDK^Emx1-KO^, *n* = 10 for BDK^M-KO^, and *n* = 6 for BDK-KO. ** *p* < 0.01 and *** *p* < 0.001 compared with control values. The difference was determined using the log-rank (Mantel–Cox) test and Dunnett’s multiple comparisons test. BCAA: branched-chain amino acids; BDK: BCKDH kinase; KO: knock-out.

Previously we showed that endurance exercise performance after training is affected by deletion of BDK in muscle and heart [19,20]. We analyzed the running capacities of BDK^Emx1-KO^ mice as well as BDK^M-KO^ and BDK-KO mice. Before training, untrained control, BDK^M-KO^, and BDK^Emx1-KO^ mice showed a similar running time on the treadmill, but in BDK-KO mice at 15–17 weeks of age, the time was significantly higher than in the other genotypes of mice (Figure 3A). After 2 weeks of training, the total running time to exhaustion increased in all mice compared to the corresponding untrained group of mice (Figure 3B). We found a considerable increase in the running time of the trained BDK-KO as well as BDK^Emx1-KO^, but not BDK^M-KO^ mice, compared to the trained control mice (*p* < 0.01) (Figure 3B).

## 4. Discussion

In astrocytes in the rodent brain, transamination of BCAAs is catalyzed by BCATm to produce glutamate and BCKAs. On the other hand, BCATc reversibly completes the BCAA cycle to form α-ketoglutarate and BCAAs by transfer of nitrogen from glutamate to BCKAs, which are in turn catalyzed by the BCKDH complex expressed in neurons [8,9]. The BCAA cycle is thought to buffer glutamate in the central nervous system to maintain normal neurological function, and thus, BCKDH must be tightly regulated by BDK. Mutations in BDK are responsible for conditions involving neurological dysfunction, such as autism, epilepsy, and intellectual disability in humans and animal models [15,16,17]. BDK-KO mice have markedly reduced BCAA levels in brain and serum, by about 70% and 60% of control mice, respectively [17]. Using a conditional gene-targeting method, we demonstrated that muscle-specific BDK-deficient mice do not exhibit any obvious neurological defects, even though BCAAs are reduced by about 30% in plasma compared to control mice [18]. Here, we generated BDK^Emx1-KO^ mice that are deficient in BDK only in cerebral cortex neurons. The mice showed only a 20% reduction in BCAAs in the forebrain, which is less than in the brain of BDK-KO mice. However, neurological abnormalities were found in BDK^Emx1-KO^ mice, especially in those fed a 5% protein diet (Figure 2), suggesting that dysregulated BCAA catabolism and decreased BCAA levels only in cerebral cortex neurons may be responsible for inducing neurological impairment.

In the present study, we also found that BDK^Emx1-KO^ mice showed increased endurance capacity following training, but not before training (Figure 3B). However, the endurance capacity of BDK^M-KO^ mice was similar to that of control mice after training (Figure 3B), suggesting that accelerating BCAA catabolism in cerebral cortex neurons, but not in muscle, enhances the effect of suppression on central fatigue by endurance exercise training. The result in BDK^M-KO^ mice disagrees with a previous study that showed decreased endurance capacity [19]. The reasons may be due to the use of different conditions of the endurance exercise method between the previous report and the present study (unlimited vs. limited running speeds and fed vs. fasted states before the test, respectively) [19,20]. The capacity test with unlimited speed used in the previous work did not allow mice to eventually catch up their speed during the test, indicating that the running distance data in the previous work may have been determined by their abilities not only in endurance but also in their ability to run fast. Here we limited the running speed to a maximum of 20 m min^−1^ to properly evaluate the endurance capacity. As a result, the running distance in the present study was about 1.2-fold higher than the previous data, suggesting that the BDK^M-KO^ mice in the present study maintain their energy sources by promoting BCAA oxidation in muscle. Furthermore, BDK-KO mice had a tremendously increased capacity compared to control mice, even before training (Figure 3). Considering the intolerance of endurance exercise in BCATm-deficient mice [11], these results suggest that the endurance capacity can be enhanced by increasing BCAA oxidation in cerebral cortex neurons and/or other regions/tissues, which may suppress central and peripheral fatigue due to running [27,28,29].

Although few reports have described the relationship between neurological dysfunction and endurance performance, our results are consistent with some mice models. γ-Aminobutyric acid receptor (GABA_B(1)_)-deficient mice, a model of epilepsy, are reported to show the enhanced locomotive activity [30]. Calmodulin M77Q mice in which wild-type calmodulin-1 replaced by a calmodulin containing a mimic of methionine sulfoxide at residue 77 show more active locomotion by the neurobehavioral test and run longer in the forced treadmill test with 20% smaller body weight than wild-type mice [31]. Overexpression of functional vesicular acetylcholine transporter gene, which leads to consequently increased cholinergic tone, also show a marked improvement in motor endurance with severe cognitive deficits, including attention deficits and dysfunction in working memory and spatial memory [32]. In addition, the endurance capacity increases in the mice overexpressing the high-affinity choline transporter (CHT), also known as solute carrier family 5 member 7 (SLC5A7), which imports extracellular choline into presynaptic terminals, thereby increasing the synthesis of acetylcholine [33]. Recently, brain glycogen stored in astrocyte has been reported to play a critical part in raising neuron energy sources by increasing lactate transport during endurance exercise, and glycogenolysis is suggested to account for a mechanism for central fatigue by inhibiting neuronal activity [34]. Although the mechanisms on how increasing BCAA oxidation enhances the endurance capacity remain unclear, this is the first report to reveal the specific cell type that causes neurological abnormalities due to BDK dysfunction, which provides a positive trend to the endurance capacity. Our findings may help generate a novel method to elevate running performance by modulating BCAA catabolism.

## Figures and Tables

**Table 1:** Tissue weights of control and BDK^Emx1-KO^ mice.

Tissue	Control			BDK^Emx1-KO^		
	**g/100 g Body Weight**					
Brain	1.43	±	0.06	1.36	±	0.06
Liver	3.66	±	0.11	3.89	±	0.07
Kidney	1.04	±	0.07	1.05	±	0.04
Heart	0.38	±	0.01	0.38	±	0.02
Adipose	6.85	±	0.37	7.52	±	0.71
Skeletal muscle	1.02	±	0.02	1.04	±	0.03

Comparison of weights of tissues relative to the body weight (g/100 g) for the control and BDK^Emx1-KO^ mice. Weights of adipose tissues and skeletal muscle correspond to the sum of the five regions of subcutaneous and visceral fat pads and the gastrocnemius, plantaris, and soleus of the mice, respectively. Values represent the mean ± SEM, *n* = 6 per group. BDK: branched-chain α-keto acid dehydrogenase (BCKDH) kinase; KO: knock-out.

**Table 2:** Brain amino acids in the control and BDK^Emx1-KO^ mice.

		Control			BDK^Emx1-KO^		
Tissue	Amino Acid	nmol/g Tissue					
Brain	Alanine	671.7	±	119.3	745.5	±	138.9
	Asparate	4840.2	±	841.9	6718.5	±	999.5
	Glysine	956.9	±	131.8	1017.8	±	143.4
	Glutamate	15,628.5	±	3287.1	16,945.9	±	2941.6
	Lysine	204.6	±	35.7	486.7	±	182.2
	Methionine	54.3	±	8.6	51.5	±	15.5
	Phenylalanine	88.4	±	17.4	89.2	±	16.5
	Proline	99.5	±	20.6	107.3	±	20.7
	Serine	1061.3	±	162.2	1203.2	±	209.7
	Threonine	336.5	±	39.4	454.6	±	82.9

Amino acid concentrations in nmol/g brain tissue of the mice. Values represent the mean ± SEM, *n* = 6 per group. BDK: BCKDH kinase; KO: knock-out.
